# The effect on colorectal cancer incidence and staging with population-based FOBT-screening in Sweden

**DOI:** 10.1186/s12889-025-22771-8

**Published:** 2025-04-26

**Authors:** Hanna Ribbing Wilén, Håkan Jonsson, Johannes Blom

**Affiliations:** 1 Department of Emergency Surgery, Karolinska University Hospital Huddinge, Stockholm, Sweden; 2 Department of Clinical Science and Education, Karolinska Institutet, Stockholm, Sweden; 3 Department of Epidemiology and Global Health, Umeå University, Umeå, Sweden; 4 Department of Surgery, Södersjukhuset, Stockholm, Sweden; 5 Karolinska University Hospital Huddinge B85, Stockholm, 141 86 Sweden

**Keywords:** Colorectal cancer screening, Fecal occult blood test, Fecal immunochemical test, Colorectal cancer incidence

## Abstract

**Aim:**

To investigate colorectal cancer (CRC) incidence and stage of disease in the population invited vs. not invited to the guaiac-based Fecal Occult Blood (gFOBT) and Fecal Immunochemical Test (FIT) colorectal cancer screening program in Stockholm-Gotland, Sweden, 2008–2021, and to estimate the incidence rate by sex and localization in the colorectum.

**Methods:**

The study cohort consisted of all 60-69-years-old residents of the Stockholm-Gotland region 2008–2012 according to the population register. Screening with biennial gFOBT was successively implemented in the region by randomly inviting birth cohorts of the target group to different year of start of screening from 2008 and replaced by FIT with cut-off level 40 µg/g in women and 80 µg/g in men for a positive test in 2015. Record linkage was made to the National Cancer Register and to the Swedish Colorectal Cancer Register (SCRCR). The age-standardized CRC incidence ratio was compared in invited and non-invited during screening and in 70-75-year-olds and assessed overall and by sex, CRC stage and localization.

**Results:**

In total, 320,989 and 151,533 individuals were invited to a first screening and FIT round, and 5,972 CRCs were diagnosed. During screening, the overall age-adjusted incidence ratio for the gFOBT- and FIT-invited compared to the non-invited was 0.99 (95% CI 0.91–1.07) and 1.03 (95% CI 0.93–1.15), respectively. Post screening, 70–75 years of age, the overall incidence rate was 12% lower among the invited than the non-invited (RR 0.88, 95% CI 0.81–0.97). During FIT screening, the incidence for stage I and proximal CRC was 38 and 23% higher than in the non-invited (RR 1.38, 95% CI 1.09–1.76 and RR 1.23, 95% CI 1.02–1.48 respectively). The incidence post screening was 22% lower regarding stage I CRC, 13% lower in women, and 17% lower for distal CRCs as compared to the non-invited (RR 95% CI 0.78 0.63–0.95, 0.87 0.76-1.00 and 0.83 0.74–0.94 respectively).

**Conclusion:**

In the Stockholm-Gotland program, the FIT screening significantly increased the incidence rate in early staged and proximal CRCs as compared to the uninvited, and the significant decrease in the overall CRC incidence post screening was mainly seen in distal, early staged CRCs in women.

**Supplementary Information:**

The online version contains supplementary material available at 10.1186/s12889-025-22771-8.

## Introduction

Colorectal cancer (CRC) is attributed to more than 900,000 deaths worldwide each year and is thus a major health concern [1]. Biennial screening with guaiac-based Fecal Occult Blood Test (gFOBT) has shown an overall 15% decrease in CRC mortality in randomized controlled studies (RCTs) and is recommended as a screening test in European and American guidelines [2–5]. It is believed that screening identifies CRC at an earlier stage than when diagnosed in symptomatic disease, and that the shift in staging is responsible for the mortality benefit. In all four RCTs a shift towards more early-staged CRCs (Dukes A) and fewer late-staged CRCs (Dukes D) was seen in the screening group as compared to the controls [2]. A meta-analysis has shown a statistically significant pooled reduction by 8% in late-stage CRC (Dukes C or D or Stage III-IV) with gFOBT screening as compared to the controls [6]. However, one systematic review revealed a poor correlation between late-stage CRC and mortality reductions in randomized studies of gFOBT screening [7].

Previous studies of CRC incidence during FOBT screening implementation are limited by the lack of a population-based setting or a control group from the same time-period and demography with repeated screening [8–13].

Moreover, changes in the CRC incidence with screening is likely different in subgroups of CRC. In a meta-analysis of gFOBT performance with colonoscopy-verified CRCs, the sensitivity for proximal cancer was significantly lower than for distal cancer (63% vs. 75%) [14]. Thus, positive screening results likely reflect CRCs located in the distal colon or rectum, and an advantage in staging distribution with screening might be limited to these malignancies [15].

The sensitivity of the Stockholm-Gotland gFOBT screening program was considerably lower in women than in men, and warranted the switch to sex-based FIT screening in 2015, which may also be reflected in the staging distributions in men and women during gFOBT and FIT screening [16].

Due to limited resources, the gFOBT screening in the Stockholm-Gotland region was implemented gradually for 60–69-year-olds in 2008–2015, with a pragmatic design of random allocation to when to start screening early, late or not by birth cohorts born between 1938 and 1954, also to facilitate an evaluation of effectiveness [17]. Half of the birth cohorts received, by definition, early invitation (2008–2012) to biennial gFOBT screening, and the remaining half received late (2013–2015) or no invitation to screening. In 2015, gFOBT was replaced by Fecal Immunochemical Test (FIT) screening. The program is intended to expand to residents up to the age of 74 years, by 2026. An evaluation of the program has estimated a CRC mortality reduction of at least 14% after a maximum of 14 years of follow-up [17]. The aim of this study was to evaluate the overall CRC incidence pattern, as well as looking at differences in sex, staging, and colorectal localization before, during and up to five years after screening cessation in the colorectal cancer screening program in Stockholm-Gotland, Sweden, 2008–2021.

## Methods

### Study population

The study population consisted of all residents born between 1938 and 1954 living in the Stockholm-Gotland region of Sweden between the years 2008 to 2012 according to the population register. In 2008, population-based colorectal cancer screening with guaiac-based Fecal Occult Blood Test (gFOBT) started to be implemented in the region by stepwise invitation of birth cohorts 1940, 1942–1954. When to start screening was randomly allocated by birth cohort and the first invitation to screening for the different birth cohorts was sent between 2008 and 2015, Fig. 1. Biennial screening invitation continued until the age of 69, i.e., 1–5 rounds, depending on age and when screening invitations started. The birth cohorts from 1938, 1939 and 1941 were never invited to screening. From 2015 and onwards, the birth cohorts born 1947–1954 were invited to FIT screening. The follow-up period for CRC was 2008–2021. Individuals with a diagnosis of CRC before start of the screening program in 2008, were excluded. No other exclusion criteria were applied.

Invitations were sent by mail from the Regional Cancer Center (RCC) of Stockholm-Gotland that coordinated the screening program and included a panel of three gFOBT tests (Hemoccult, Beckman Coulter, U.S.A.) with information on CRC screening and instructions on how to perform the test. The FIT test kit included the invitation and one FIT test tube (OC Sensor, Eiken, Japan). The participants were instructed to note the date of the sample and to send the test in a prepaid envelope to the laboratory as soon as possible. The gFOBT samples were visually inspected by laboratory personnel. A sample was classified as positive if it exhibited an oxidase reaction in at least one of the three samples. In October 2015, the program changed to Fecal Immunochemical Test (FIT) with sex-based cut-off levels. Women with FIT ≥ 40 µg Hemoglobin/g and men with FIT ≥ 80 µg/g were considered FIT positive. A new kit was sent in case of an unanalyzable result, and a reminder was sent after 8 weeks, in case of non-response.

All participants with a positive test were offered colonoscopy at the nearest of five contracted endoscopy units. Those with a negative test were advised to consult the primary health care provider in case of bowel symptoms. There was no other CRC screening offered to the residents of the region.

### Data sources

All residents in Sweden have a unique personal ID number assigned at birth and used in all contacts with authorities and health care units and can thus be linked to various registers. The data on residents born between 1938 and 1954 registered in the Stockholm-Gotland region and information on emigration were retrieved from Statistics Sweden, the national population registry. Record linkage was made to the screening register at RCC containing data on screening status and invitations, gFOBT/FIT- and colonoscopy results. Data was further linked to the National Cancer Register, 1958–2020, where reporting of cancer cases is mandatory by law, for information on CRC diagnosis and date of diagnosis, and to the Swedish Colorectal Cancer Register (SCRCR) between the years 2008–2021 that comprises information on CRC diagnosis for the remaining year 2021, CRC staging and localization. The SCRCR has a coverage of 99% and an overall validity of 90% compared to hospital patient records [18]. The National Cancer Register has a coverage of 96% based on data from 1998 [19]. The data sources and linkage procedures are described in more detail by Blom et al. [17].

### Colorectal cancers

The CRC diagnosis included the International Classification of Diseases 7 (ICD-7) code 153.X (malignant neoplasm of large intestine, except rectum) or 154.0 (malignant neoplasm of rectum), excluding the codes C24; 091 (neuroendocrine tumor), 093 (lymphoma), 094 (adenoma), 144 (squamous cell carcinoma) and 793 (gastrointestinal stroma tumor**).** CRCs were classified according to the TNM system in stage I-IV: stage I (T1-2, N0, M0), stage II (T3-4, N0, M0), stage III (T1-4, N1-2, M0) and stage IV (M1) [20]. Early CRC was defined as stage I-II and late CRC as stage III-IV. During the study period the 6th and 7th edition of the TNM Classification of Malignant Tumors was used in Sweden, and the main T, N, and M categories as well as the CRC stages I-IV remained the same between editions [21]. The localization of CRCs was grouped into proximal CRC (caecum to splenic flexure) and distal CRC (descending colon to rectum).

### Statistics

The study cohort consisted of individuals invited to and not invited to screening. The follow-up for each individual started in 2008 and continued until 2021, death, or emigration, whichever came first, and was divided into four exposure categories: before first invitation including never invited, after first invitation to screening with gFOBT, after first invitation to screening with FIT, and post screening, i.e., after the last screening round. Number of person-years and incident CRC cases were calculated by attained age and exposure category; hence the same individual could belong to several exposure categories. The last invitation to screening occurred when the upper age limit for the program was reached (68 or 69 years), or when a CRC or an advanced adenoma that required polyp surveillance was detected, since that individual was admitted to surgery or the polyp surveillance program and not re-invited to the screening program, or if an invited individual migrated from the region. After the date of the last screening invitation which were either gFOBT or FIT, the exposure category was kept during the following two years of follow-up, corresponding to the biennial screening interval. Thus, the last screening round included up to 71-year-olds, and CRCs detected during the period two years after the last invitation was classified as a CRC in those invited. As an example, the birth year cohort of 1950 were not yet invited in 2008–2009, invited to gFOBT 2010–2014, invited to FIT 2016–2018 and post screening 2020-21. The incidence in previously invited age groups were compared with never invited of the same age and continued until the age of 75, since the effect of screening on the incidence is likely to diminish a few years after screening cessation.

Those invited to FIT was kept as a separate exposure category from 2015. Due to the ageing of the study cohort and the biennial invitation scheme, population birth cohorts for the years 1938–1946 were never invited to the FIT program and population birth cohorts for the years 1947–1954 contributed relatively few person-years at ages 60–61. Therefore, the CRC incidence of invited and non-invited in the FIT program was estimated for the age-range 62–69 years.

The CRC incidence rate was assessed according to invitational status, regardless of participation, and whether the CRC was screening-detected or an interval CRC (a CRC not detected at screening and diagnosed between screening rounds) and analyzed by stage, sex, and localization in colorectum. The CRC incidence was assessed by year of attained age. However, due to a small number of CRC cases and invited at the age 61, 63 and 67, ages with less than 5,000 person-years were omitted in Fig. 2 (Additional file 1).

Cumulative incidence was calculated for the three categories of individuals invited to gFOBT screening in ages 60–69, individuals invited to FIT screening in ages 62–69, and post-screening individuals aged 70–75, and compared to the cumulative incidence for not yet or never invited in the corresponding age intervals. Due to differences in age distribution, age-standardized incidence ratio (SIR), i.e., with adjustments of age, was calculated with the not yet or never invited category as reference [22]. Similarly, SIR was calculated for FIT vs. gFOBT. The CRCs diagnosed more than two years after the last screening invitation in the age category 60–69-year-olds, e.g., individuals in the polyp surveillance program, were not included among the gFOBT/FIT invited. In addition, a sensitivity analysis was conducted where all 60–69 years-olds, e.g. in the polyp surveillance, were also included among those invited to gFOBT and FIT screening. Post screening, at the age of 70–75, all previously invited to screening was compared to never invited. A p-value of < 0.05 was considered statistically significant, and 95% Confidence Intervals (CI) were calculated. For all statistical analyses, the R statistical software version 4.2.2 was used (R project for statistical computing, URL https://www.R-project.org/).

## Results

**Fig. 1:**
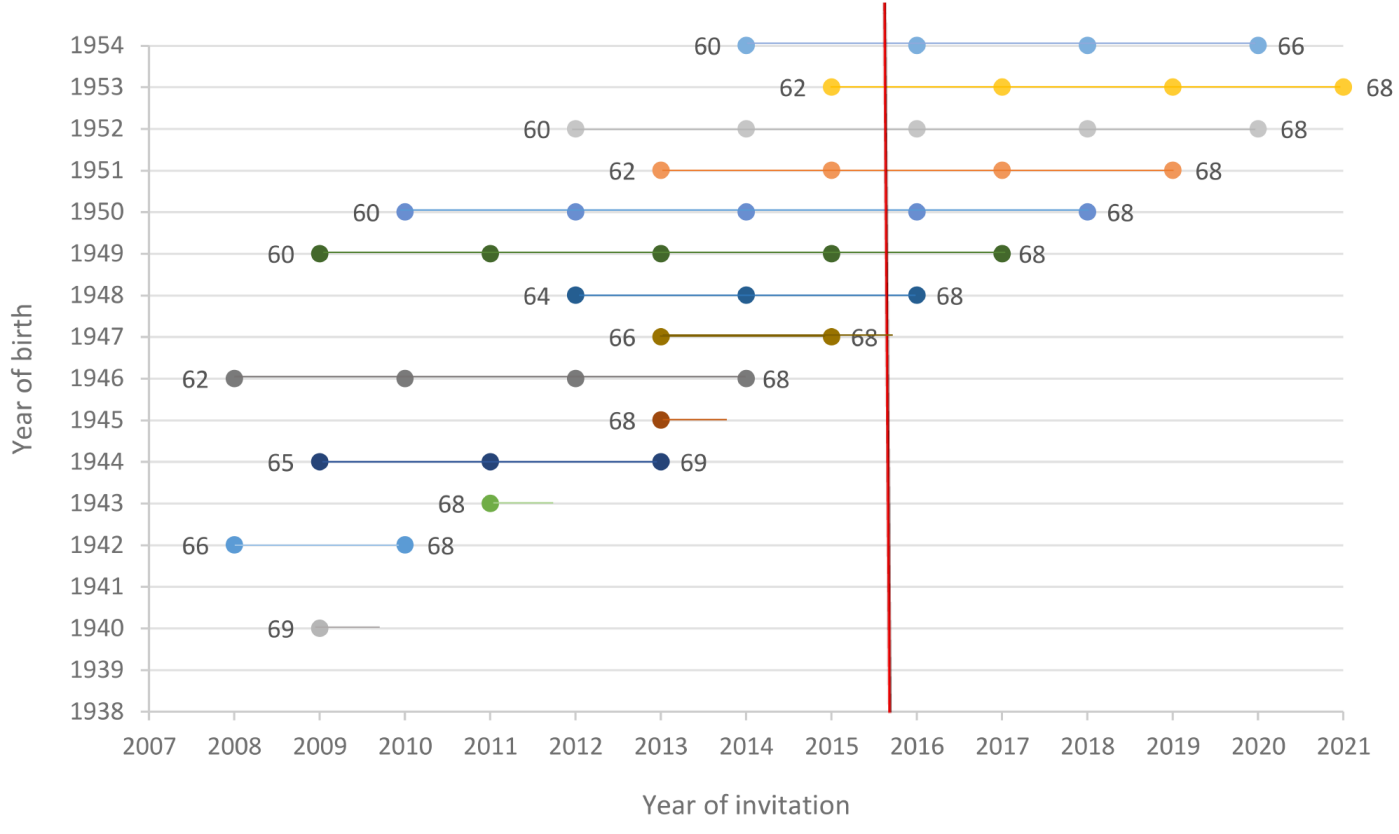
Invitation schedule to gFOBT and FIT screening by birth year and attained age in the Stockholm-Gotland regional program 2008–2021. The vertical red line marks the shift to FIT screening in October 2015

Between 2008 and 2012, 392,161 individuals born between 1938 and 1954 were registered in the Stockholm-Gotland region. The study cohort consisted of 390,040 residents after excluding 2,121 individuals with a diagnosis of CRC before 2008. In total, 320,989 and 151,533 were invited to a first screening and a first FIT screening round respectively. Of the FIT invited, 2,199 were screening naïve. In the cohort, 69,051 were never invited to screening. The median follow-up time was 14 years. The invitation schedule and number of screening round invitations in each population birth cohort is illustrated in Fig. 1. The number of individuals invited to a first screening round and first FIT screening at each attained age is listed in Additional file 1, and the size of each population birth cohort in Additional file 2.

The mean follow-up-time in the exposure categories before or no invitation, invited to gFOBT, invited to FIT, and post screening was 4.9, 4.0, 3.9 and 5.4 years, respectively. Overall, 5,972 CRCs were diagnosed. Within two years from the last gFOBT and FIT screening invitation, 1,572 and 846 CRCs in 60–69-year-olds, and 46 and 31 in 70–71-year-olds were diagnosed. There were 970 CRCs in 60-69-year-olds never or not yet invited to screening. Furthermore, 1,762 CRCs were diagnosed post screening as compared to 653 in those never invited to screening and of the same age (Table 1). There were 92 (1.5%) CRCs diagnosed after two years from the last invitation to screening in the age category 60-69-year-olds during 57,375 person-years of follow up, corresponding to the CRCs detected in the polyp surveillance program or in those that migrated from the region. These CRCs were not included among the post screening analyses of 70–75-year-olds.

**Table 1:** Accumulated person-years and colorectal cancer characteristics in each exposure category of the 390,040 residents invited and not invited to the Stockholm-Gotland screening program

	Before screening or no screening, *n* (%)	Invited to gFOBT screening, *n* (%)	Invited to FIT screening, *n* (%)	Post screening, invited* n (%)	Post screening, uninvited** n (%)
Person-years	794,960 (100)	1,232,493 (100)	568,683 (100)	923,471 (100)	299,759 (100)
Men	388,200 (49)	596,818 (48)	275,415 (48)	437,681 (47)	142,477 (48)
Women	406,760 (51)	635,674 (52)	293,268 (52)	485,790 (53)	157,282 (52)
CRC	970 (100)	1572 (100)	846 (100)	1762 (100)	653 (100)
CRC, sex					
Men	562 (58)	891 (57)	483 (57)	978 (56)	358 (55)
Women	408 (42)	681 (43)	363 (43)	784 (44)	295 (45)
CRC stage					
I	170 (18)	337 (21)	213 (25)	330 (19)	138 (21)
II	244 (25)	390 (25)	175 (21)	448 (25)	171 (26)
III-IV	497 (51)	743 (47)	396 (47)	901 (51)	311 (48)
Unknown	59 (6)	102 (6)	62 (7)	83 (5)	33 (5)
CRC localization					
Proximal	286 (29)	527 (34)	296 (35)	736 (42)	250 (38)
Distal	651 (67)	991 (63)	518 (61)	976 (55)	380 (58)
Unknown	33 (3)	54 (3)	32 (4)	50 (3)	23 (4)

CRC = colorectal cancer. gFOBT = guaiac-based fecal occult blood test. FIT = Fecal immunochemical test. Post screening = age 70–75. Stage I = T1-T2 CRC with no regional lymph node metastases. Stage II = T3-T4 CRC with no regional lymph metastases. Stage III = CRC with regional lymph metastases. Stage IV = CRC with distant metastases. Proximal localization = caecum to splenic flexure, distal localization = descending colon to rectum

*) The last screening invitation occurs at the age 68 or 69, or when a CRC or high-risk adenoma is diagnosed in invitees aged < 69 since they are not reinvited to the program. In this column the CRCs in 60–69-year-olds are not included, only CRCs in individuals at the age of 70–75. The CRCs diagnosed within two years from last screening invitation are considered attributed to the screening due to the biennial invitation scheme and thus not included

**) Post screening, uninvited, refers to the 70–75-year-olds that were never invited to screening at the age 60–69

**Fig. 2:**
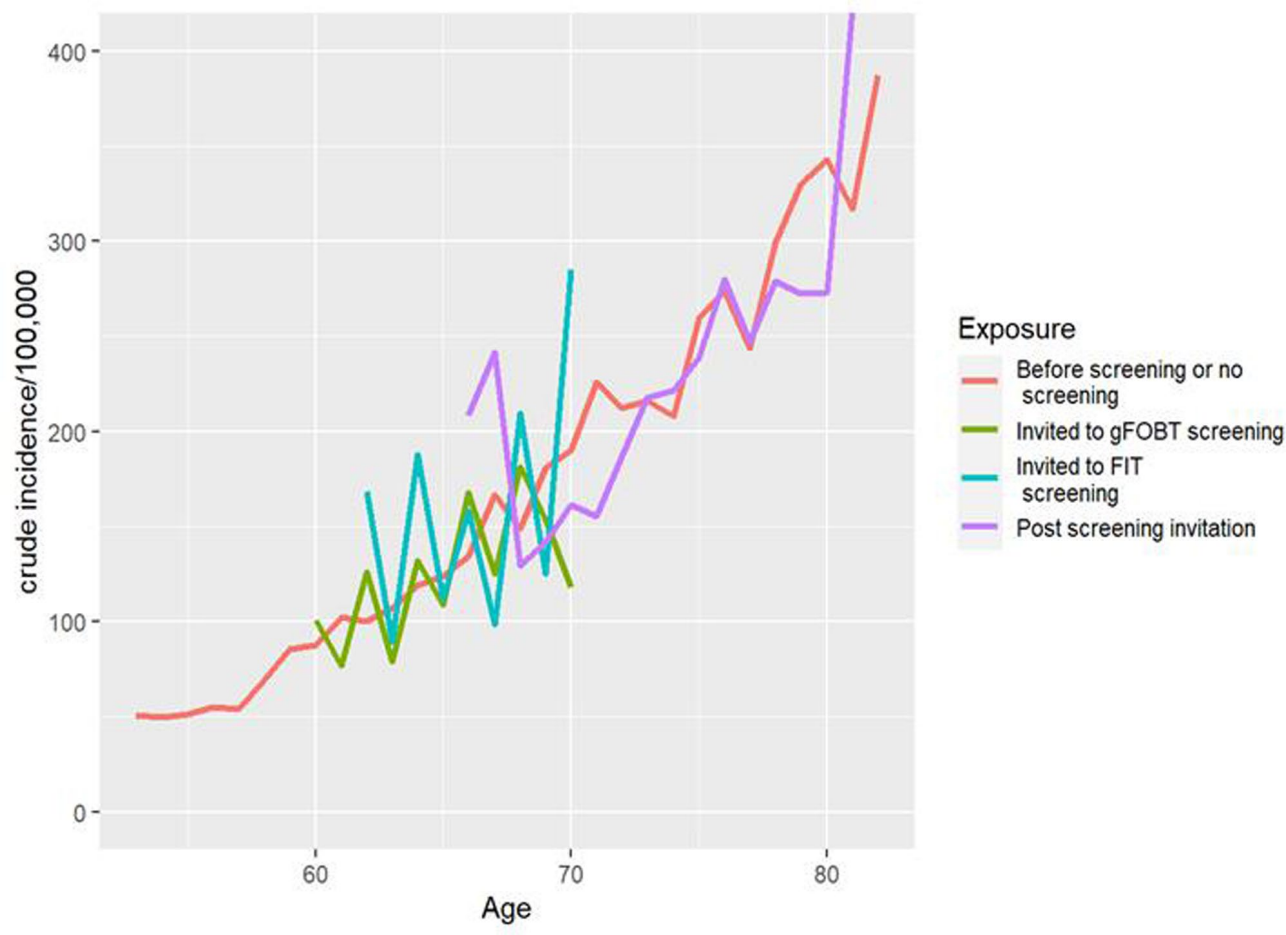
CRC incidence per 100,000 person-years and attained age in those invited and not invited to screening The incidence is plotted for ages with > 5000 person-years of follow-up. Red line = Incidence in individuals not (yet) invited to screening. Green line = Incidence in those invited to gFOBT screening (from 2008). Blue line = incidence in those invited to FIT screening (from 2015). Purple line = Incidence two years after the last invitation to screening. CRC = colorectal cancer. gFOBT = guaiac-based fecal occult blood test. FIT = Fecal immunochemical test

The CRC incidence rate per person-years for each attained age is displayed in Fig. 2 for individuals invited to FIT and gFOBT and for the post screening and non-invited categories. The cumulative CRC incidence rate per 100,000 person-years was 122, 128, 149, 191 and 218 for non-invited or not yet invited, gFOBT invited 60–69 years-olds, FIT invited 62-69-year-olds, post screening invited and post screening non-invited individuals 70–75 years-olds, respectively.

The age-adjusted incidence ratio (SIR) for the gFOBT- and FIT-invited compared to the non-invited was similar (RR 0.99 95% CI 0.91–1.07 and 1.03 95% CI 0.93–1.15). However, there was a 12% decrease (RR 0.88, 95% CI 0.81–0.97) in the incidence among previously invited 70–75-year-olds, as compared to the non-invited of the same age (Table 2). In the sensitivity analysis, all 60-69-year-olds in the polyp surveillance were included among the gFOBT and FIT invited, and this did not significantly change the results (RR 0.99 95% CI 0.91–1.08 and 1.03 95% CI 0.92–1.14).

The decreased age-adjusted incidence ratio post screening as compared to the non-invited remained in women (RR 0.87, 95% CI 0.76-1.00) but not significantly in men. Regarding CRC stage, there was an increase in stage I CRC of 38% (RR 1.38, 95% CI 1.09–1.76) for the FIT-invited, and a 22% decrease (RR 0.78, 95% CI 0.63–0.95) in incidence post screening as compared to the non-invited. The incidence in late-stage (III and IV) CRC was similar in invited and non-invited both during and after screening. The FIT screening increased the proximal CRC incidence by 23% (RR 1.23, 95% CI 1.02–1.48), but the proximal CRC incidence was not decreased post screening (RR 0.98, 95% CI 0.84–1.13). There was no significant difference during the gFOBT screening in subgroups of CRC as compared to the non-invited. Distally located CRCs decreased with 17% post screening (RR 0.83, 95% CI 0.74–0.94) (Table 2).

**Table 2:** Age-standardized CRC incidence ratio in invited to gFOBT and FIT screening, respectively, vs. non-invited in the Stockholm-Gotland program

	Age-standardized rate ratio (95% CI)			
	Non-invited	gFOBT	FIT	Post screening
Overall	1	0.99 (0.91–1.07)	1.03 (0.93–1.15)	0.88 (0.81–0.97)
Gender				
Men	1	0.99 (0.88–1.10)	1.05 (0.91–1.21)	0.89 (0.79–1.01)
Women	1	0.99 (0.87–1.12)	1.01 (0.86–1.19)	0.87 (0.76-1.00)
CRC stage				
I	1	1.17 (0.96–1.41)	1.38 (1.09–1.76)	0.78 (0.63–0.95)
II	1	0.98 (0.83–1.15)	0.84 (0.68–1.05)	0.87 (0.73–1.04)
III-IV	1	0.92 (0.81–1.03)	0.97 (0.83–1.13)	0.94 (0.83–1.07)
CRC localization				
Proximal	1	1.13 (0.97–1.31)	1.23 (1.02–1.48)	0.98 (0.84–1.13)
Distal	1	0.92 (0.83–1.02)	0.93 (0.81–1.06)	0.83 (0.74–0.94)

gFOBT = guaiac-based Fecal Occult Blood Test. FIT = Fecal immunochemical test. The cumulative incidence for gFOBT screening is calculated for age 60–69 and that of FIT for age 62–69. Post screening refers to age 70–75. Distal colon = descending to rectum. Proximal colon = Caecum to splenic flexure. Stage I = T1-T2 CRC with no regional lymph node metastases. Stage II = T3-T4 CRC with no regional lymph node metastases. Stage III = CRC with regional lymph node metastases. Stage IV = CRC with distant metastases

Comparing FIT and gFOBT screening, the differences in incidences were non-significant; the overall age-adjusted incidence ratio was 1.03 (95% CI 0.94–1.12). For stage I CRC, the incidence ratio was 1.19% (95% CI 0.99–1.42) in FIT as compared to gFOBT screening. Moreover, the overall incidence in FIT-invited men was non-significantly higher than in gFOBT-invited men (RR 1.03, 95% CI 0.92–1.16). The difference between FIT and gFOBT incidences of stage II, III-IV, proximal and distal localization and in women was non-significant (RR 0.85 95% CI 0.70–1.02, RR 1.02 95% CI 0.90–1.16, RR 1.04 95% CI 0.90–1.21, RR 1.02 95% CI 0.91–1.14, and RR 1.02 95% CI 0.89–1.16, respectively).

## Discussion

This evaluation of the population-based screening program in Stockholm-Gotland, Sweden, demonstrated a similar overall CRC incidence among those invited to the screening as compared to the non-invited, and a decreased incidence after screening cessation. This change in post screening incidence was significant for early-staged CRC and CRCs in women and differed by colorectal localization. However, we did not find a significant change in incidence in subgroups during the gFOBT program, nor a decrease in late-stage CRC from the screening program.

Several previous studies have shown an increased CRC incidence when introducing population-based FIT screening, due to both incidence and prevalence screening in the first round, and thereafter a return to or decrease below pre-screening levels in subsequent rounds [12, 23, 24]. At long-term follow-up of the randomized gFOBT screening trial in Minnesota, there was a marked decline in incidence with multiple screening rounds as compared to controls [25].

In our study, we did not find a significant change in the incidence among those invited to the gFOBT program. However, the invited age cohorts in the gFOBT screening consisted of both prevalent and incident screening rounds for each attained age (except the 60-years-olds), hence the net effect could be an unchanged incidence. Apart from this, approximately 40% of the gFOBT invitees did not participate in screening and could therefore not contribute to an increased CRC incidence [26]. Furthermore, gFOBT, as compared to FIT, has a lower sensitivity for advanced adenoma; 10–15% vs. 25–30%, hence a large decline is not expected in CRC incidence post screening because of polypectomy in a gFOBT screened population [27–30]. In the Minnesota trial, the cumulative gFOBT positivity was approximately 30%, so the large decrease in incidence could partly be explained by the large number of colonoscopies (and polypectomies) performed in the intervention group. The positivity rate in the Stockholm-Gotland gFOBT program was approximately 2% [16].

The gFOBT sensitivity for CRC is stage-dependent, with reported sensitivity rates of Dukes A 89%, Dukes B 79%, Dukes C 72%, and Dukes D 48% [15]. A meta-analysis of FIT studies (cut-off-levels between 10 and 20 µg/g) estimated FIT sensitivity to 73% for stage I CRC, 80% for stage II, 82% for stage III, 79% for stage IV [31]. The overall CRC sensitivity in the Stockholm-Gotland FIT program with cut-off levels 40 µg/g and 80 µg/g in women and men was estimated at 65%, as compared to 40% in the gFOBT program [16, 32]. The shift to FIT in the program generated an increased participation rate (69%), and a higher sensitivity for advanced adenomas and CRC, which contributed to the net increase in CRC incidence in subgroups of early-staged and proximal CRCs during screening and a decrease post screening due to the preventive effect of polypectomy. The decreased incidence post screening after an increase during screening, as compared to the non-invited, was only seen in stage I CRCs, probably due to the earlier detection of CRC with screening leading to a compensatory drop after screening cessation.

The 14% reduction in CRC mortality recently reported from the Stockholm-Gotland screening program is most likely explained by a shift from late to early-stage CRC [17]. However, in the present study we did not a see a decreased incidence of stage III-IV CRC among the invited. Since the mortality is low in stage I CRC, the reduced stage I incidence post screening could not explain the reduced mortality [33]. However, the invited included both the non-participants as well as prevalence screening rounds which could balance out a favorable stage shift in the participants. In the Dutch screening program, the incidence of late-stage CRC increased at screening implementation followed by a decrease when compared to the incidence rates prior to screening [8]. Moreover, given the low number of CRCs stage III and IV in the present study, they were combined and categorized into late-stage (III-IV). There might be a stage shift among the invited from IV to III or in subclassification of T stages that we were not able to detect albeit important for the prognosis [34]. In the previously cited Danish study, there was an increased incidence of stage I-III CRC and no significant difference in stage IV CRC in the invited as compared to non-invited, but this study included only the prevalence round [12].

On the other hand, it is not certain that a favorable stage shift due to screening directly translates into a disease specific mortality reduction, because this is dependent on the difference in survival between the early and late stages and the proportion of cancers that the screening is able to shift to earlier stages, and it also assumes that the late stage cancers among the invited have the same survival as the late staged cancers among the non-invited [35]. Invitation to screening could raise the awareness of the disease and make the invited individuals more prone to seek health care and to life style changes, e.g., regarding smoking, than the non-invited, potentially affecting the disease mortality other than as a shift in stage [36].

During the FIT screening, the incidence of proximal CRC increased. Proximal CRCs are more common in women, and the Stockholm-Gotland program applies lower cut-off-levels in women than in men, which might explain the increased incidence with FIT screening and the decreased incidence in women post screening, although gFOBT and FIT performs worse in proximal CRCs as compared to distal in sex-uniform screening [14, 32]. The increased incidence of early-stage proximal CRCs in women could have contributed to the decreased mortality seen in the screening the program, since proximal CRCs confers a worse prognosis and are diagnosed clinically at a late stage [37]. The overall incidence of proximal CRC was not decreased post screening. However, only population birth cohorts from 1948 to 51 (and part of 1947 cohort) were screened with FIT and reached post screening age at end of follow-up, hence gFOBT screening was over-represented in this group. Post screening, a significant decrease was seen only for distal localization reflecting the higher sensitivity for distal CRC of both gFOBT and FIT and the higher rate of distal CRC in men [14].

The strengths of this study were the evaluation of a large population-based CRC screening program shifting from gFOBT to FIT screening, the linkage with individual data to validated cancer registers with low number of missing data enabling analyzation of CRC subgroups, and the assessment of CRC incidence of invited and non-invited individuals within the same time-period and in the same region. Moreover, evaluation of the invitation to screening precluded the self-selection bias from healthy participants.

Nevertheless, this study has limitations. The screening program implementation was made by randomization of population birth cohorts into early, late or no screening invitation rather than individual randomization, making the comparisons between invited and non-invited biased by age. This problem was overcome when comparing age-adjusted incidence ratios, but we were unable to assess the incidence change over time when initiating screening and by subsequent screening rounds. Furthermore, we did not analyze stage III and IV CRCs separately due to a low number of cases, hence not capture any change in incidence in between the late-stage CRCs as discussed above. Moreover, the post screening comparisons of incidence were, due to the recent shift to FIT screening, dominated by gFOBT screened individuals, and additional studies are needed to fully address the effect of FIT in 70-75-year-olds, especially since the shift to FIT increased the participation rate by 12% [38]. In addition, since the FIT-program applies sex-specific cut-off levels for a positive FIT and a higher cut-off level than many other European screening programs, the results could not directly be generalized to other FIT screening strategies with lower or sex-uniform cut-offs.

In conclusion, during the FIT screening in the population-based screening program of Stockholm-Gotland, Sweden, the CRC incidence for early-staged and proximal CRCs significantly increased, and the overall decrease post screening was mainly seen in distal, early staged CRCs in women as compared to the non-invited. The full effect of sex-based FIT screening on the incidence post screening needs further evaluation.

## Electronic supplementary material

Below is the link to the electronic supplementary material.

Supplementary Material 1

Supplementary Material 2

## Data Availability

Data is provided within the manuscript and supplementary information files. Access to underlying research material can be obtained from corresponding author Hanna Ribbing Wilén, within the framework of the Swedish data protection legislation and any required permissions from authorities.
