# Supplementary material for: The effect on colorectal cancer incidence and staging with population-based FOBT-screening in Sweden
Source: BMC Public Health. 2025 Apr 26;25:1557. doi: 10.1186/s12889-025-22771-8 (PMC12032652; doi:10.1186/s12889-025-22771-8)
Supplement: Supplementary file 1 — Supplementary Material 1 [file 12889_2025_22771_MOESM1_ESM.docx]

Additional file 1. Number of invited to the first screening and first FIT screening round by attained age in the Stockholm-Gotland screening program.

| Age | Invited to first screening,  n (%) | Invited to first FIT screening,  n (%) |
| --- | --- | --- |
| 60 | 92,374 (29) | - |
| 61 | 106 (0.03) | - |
| 62 | 70,324 (22) | 23,483 (15) |
| 63 | 109 (0.03) | 9 (0.006) |
| 64 | 25,709 (8) | 43,069 (28) |
| 65 | 24,249 (8) | 62 (0.04) |
| 66 | 44,531 (14) | 41,694 (28) |
| 67 | 868 (0.3) | 73 (0.05) |
| 68 | 47,016 (15) | 43,003 (28) |
| 69 | 15,673 (5) | 103 (0.07) |
| 70 | 28 (0.009) | 36 (0.02) |
| 71 | 2 (-) | 1 (-) |
| Total | 320,989 (100) | 151,533 (100) |
