# Supplementary material for: The effect on colorectal cancer incidence and staging with population-based FOBT-screening in Sweden
Source: BMC Public Health. 2025 Apr 26;25:1557. doi: 10.1186/s12889-025-22771-8 (PMC12032652; doi:10.1186/s12889-025-22771-8)
Supplement: Supplementary file 2 — Supplementary Material 2 [file 12889_2025_22771_MOESM2_ESM.docx]

Additional file 2. Birth cohort sizes in the Stockholm-Gotland region 2008-2012.

| Birth cohort | Invited to screening,  n (%) |
| --- | --- |
| 1938 | 15,222 (4) |
| 1939 | 16,214 (4) |
| 1940 | 16,671 (4) |
| 1941 | 18,342 (5) |
| 1942 | 21,288 (5) |
| 1943 | 23,842 (6) |
| 1944 | 25,874 (7) |
| 1945 | 26,506 (7) |
| 1946 | 26,608 (7) |
| 1947 | 26,566 (7) |
| 1948 | 25,915 (7) |
| 1949 | 24,865 (6) |
| 1950 | 24,595 (6) |
| 1951 | 23,738 (6) |
| 1952 | 24,485 (6) |
| 1953 | 24,669 (6) |
| 1954 | 24,640 (6) |
| 1938-54 | 390,040 (100) |
